# S31. BASIC SENSE OF SELF IN YOUTH AT HIGH RISK FOR DEVELOPING SCHIZOPHRENIA

**DOI:** 10.1093/schbul/sby018.818

**Published:** 2018-04-01

**Authors:** Hadar Hazan, Richard Linscott, Elaine Reese

**Affiliations:** University of Otago

## Abstract

**Background:**

Phenomenological researchers argue that schizophrenia is first and foremost a disorder of the basic sense of self (also known as ipsity, minimal or core self), that is, of the immediate, pre-reflective, embodied sense of being immersed in the world. According to the self-disorder model, impairment of the basic sense of self precedes clinical symptoms and is independent of them. Therefore, we postulated that youth at high psychometric risk for developing schizophrenia would present an impairment in their basic sense of self, as measured by levels of ego strength, basic symptoms, and pronoun usage.

**Methods:**

Eighty undergraduate students aged 19–22 years (M = 20.83 years, SD = 1.28 years) completed the Schizotypal Personality Questionnaire (SPQ), Ego Strengths Questionnaire (ESQ), a self-report version of Schizophrenia Proneness Instrument (SPI-A), and four written narratives about personal and fictional experiences. Based on the SPQ scores, participants were allocated to either control (at or below the 84th percentile on all three subscales) or study group (above the 90th percentile on at least one subscales). To obtain the linguistic dimension of the pronouns usage in the written narratives, the essays were subjected to Linguistic Inquiry and Word Count (LIWC).

**Results:**

Compared to the control group, the high-risk group presented lower levels of ego strength, higher levels of basic symptoms, and used more personal pronouns and the they pronoun in narratives. Self-report on the SPI-A and ESQ correlated significantly with the objective lexical pattern of pronoun use: Lower ego strength correlated with greater use of they and more self-reported basic symptoms correlated with greater use of pronouns overall, personal pronouns, and the pronouns she and they. Ego strength had the most predictive power for group membership

**Discussion:**

In line with the hypotheses, there were significant differences between the schizotypy and the control groups in objective and subjective measures of basic sense of self. Subjective measures indicated a lower level of ego strength and higher levels of basic symptoms for the schizotypy group, as compared with the control group. Objective measures revealed a different lexical pattern with higher use of third-person and personal pronouns for the schizotypy group, as compared with the control group. Subjective (basic symptoms and ego strength) and objective measures significantly correlated with each other (pronoun use). Nevertheless, it is only the level of cognitive-perceptive disturbances that best predicted membership of the schizotypy group. Taken together, these results indicate a weak sense of basic self, namely a self-disorder, in a nonclinical population. Detection of self-disorder in the premorbid and prodrome stages of schizophrenia, paired with a suitable intervention, can help to prevent or at least minimize, the eruption of its active stage. In the future, it needs to be determined how these measures of self-disorder in non-clinical population can predict transition to schizophrenia and to other psychotic disorders. Furthermore, it would be valuable to test the distributions of measures of self-disorder in younger population from a more diverse background, such as high school students from a different socio-economic background.

Lastly, it is possible to conclude that some impairment in the basic sense of self does exist in schizotypy. This is apparent across the measures of self-disorder and suggests that there is a core feature that distinguishes schizotypes from non-schizotypes.

